# Direct In Vivo Analysis of CBD- and THC-Acid Cannabinoids and Classification of Cannabis Cultivars Using SpiderMass

**DOI:** 10.3390/metabo12060480

**Published:** 2022-05-26

**Authors:** Nina Ogrinc, Serge Schneider, Adèle Bourmaud, Nicolas Gengler, Michel Salzet, Isabelle Fournier

**Affiliations:** 1 Laboratoire Protéomique, Réponse Inflammatoire et Spectrométrie de Masse (PRISM), Inserm U1192, Université de Lille, F-59000 Lille, France; nina.ogrinc@univ-lille.fr (N.O.); michel.salzet@univ-lille.fr (M.S.); 2 Service de Toxicologie Analytique–Chimie Pharmaceutique, Laboratoire National de Santé (LNS), Dudelange, L-3555 Luxembourg, Luxembourg; serge.schneider@lns.etat.lu (S.S.); adele.bourmaud@lns.etat.lu (A.B.); nicolas.gengler@lns.etat.lu (N.G.); 3 Institut Universitaire de France (IUF), F-75000 Paris, France

**Keywords:** water-assisted laser desorption/ionization, SpiderMass, cannabinoids, mass spectrometry, plants, in vivo

## Abstract

In recent years, cannabis and hemp-based products have become increasingly popular for recreational use, edibles, beverages, health care products, and medicines. The rapid detection and differentiation of phytocannabinoids is, therefore, essential to assess the potency and the therapeutic and nutritional values of cannabis cultivars. Here, we implemented SpiderMass technology for in vivo detection of cannabidiolic acid (CBDA) and ∆^9^-tetrahydrocannabinolicacid (∆^9^-THCA), and other endogenous organic plant compounds, to access distribution gradients within the plants and differentiate between cultivars. The SpiderMass system is composed of an IR-laser handheld microsampling probe connected to a mass spectrometer through a transfer tube. The analysis was performed on different plant organs from freshly cultivated cannabis plants in only a few seconds. SpiderMass analysis easily discriminated the two acid phytocannabinoid isomers via MS/MS, and the built statistical models differentiated between four cannabis cultivars. Different abundancies of the two acid phytocannabinoids were found along the plant as well as between different cultivars. Overall, these results introduce direct analysis by SpiderMass as a compelling analytical alternative for rapid hemp analysis.

## 1. Introduction

The hemp plant or cannabis (*Cannabis sativa* L. and subspecies *indica*) has been cultivated for recreational, medicinal, and industrial purposes since ancient history. The popularity of cannabis has increased over the last few years due to its unparalleled versatility of use, particularly for medicinal purposes.

Cannabinoids are synthesized from geranyl pyrophosphate and olivetolic acid and they are always present in the carboxylated “acid” form of the plants, the most abundant being ∆^9^-tetrahydrocannabinolic acid (∆^9^-THCA) and cannabidiolic acid (CBDA) [1]. Out of many, “potency” remains the most frequently measured feature in cannabis and refers to the percentage of THC and CBD (the decarboxylated forms of THCA and CBDA) present in samples. The ratios of THCA and CBDA from fresh plants can therefore be directly implied for the potency of specific cultivars. These cannabinoids display remarkable similarities as structural isomers but differ in their pharmacological properties. Indeed, only THC shows psychotropic effects while CBD has anti-inflammatory effects [2,3]. In addition, potency can also be reflected as a function of other plant constituents, mainly terpenes and flavonoids, present in different ratios in various cannabis strains or “cultivars”. It is, therefore, important to also consider additional chemical characterization of the cultivar composition in order to better decipher the pharmacological effects associated with cannabis products [2,4]. Various methods have, therefore, been developed around the analysis of cannabis. In targeted analysis of cannabinoids, the most commonly used techniques include gas chromatography (GC) with a flame ionization detector (FID) or coupled with mass spectrometry (MS), liquid chromatography (LC) with UV detection or coupled with MS, as well as other techniques such as thin layer chromatography (TLC), nuclear magnetic resonance (NMR), and near-infrared (NIR) spectroscopy [5]. However, to date, the only official methods for the quantification of cannabinoids remain HPLC-UV and GC-FID [6].

Although not an official technique, mass spectrometry (MS) has been playing an important role in the detection of CBDA, THCA, and their decarboxylated forms. However, it is not possible to detect CBDA and THCA via GC-MS analysis from fresh plant samples. Increased temperatures, indeed, trigger decarboxylation and other reactions prevent their detection in the acid forms [2]. On the other hand, LC-MS, where decarboxylation is avoided, has been used for the quantitative detection of THCA and CBDA along with other cannabinoids from dried cannabis [7], hemp seed batches, food and feed products [8,9], seized street cannabis samples and medicines [10], as well as hemp consumer products such as oils, plant material, creams, and cosmetics [11]. An interesting kinetic study on the thermal degradation of 14 phytocannabinoids was performed by low resolution mass spectrometry (LRMS) targeted analysis from dried plant material [12]. The aforementioned examples demonstrate the usefulness of LC-MS-based techniques for the quantification of cannabinoids, but this approach requires multistep sample preparation (drying, grinding, homogenization, extraction, etc.) [13] leading to a loss in the spatial information. Furthermore, the analysis cannot be performed rapidly on fresh plant samples directly in situ and in vivo.

Ambient ionization mass spectrometry (AIMS) techniques, therefore, pose a significantly less expensive alternative as they enable direct sample analysis without any sample preparation. For example, desorption atmospheric pressure photoionization mass spectrometry (DAPPI-MS) has been used for the direct analysis of THC and CBD in marijuana samples confiscated by the police. Thanks to the formation of molecular ions, the discrimination of THC and CBD has been made possible via MS/MS analysis [14]. Alternatively, paper spray MS has been used for the detection of THC and CBD in commercial oils through the use of a Ag(I)-impregnated paper [15]. Direct analysis or “swabbing” of cannabis leaves has also been evaluated using DESI-MS [16] and an atmospheric solids analysis probe (ASAP) using the positive ion mode [2]. However, most of these techniques can only be foreseen as preliminary screening techniques for targeted compounds in hemp and marijuana samples [2]. Indeed, the clear differentiation of THC- and CBD-acid derivatives in combination with other plant molecules remains challenging and incomplete. Recent reports suggest that it is possible to differentiate between the cannabinoid isomers by accurate MS/MS fragmentation patterns in the negative ion mode following specific guidelines [17].

In this work, we present an analytical alternative, water-assisted laser desorption ionization mass spectrometry (WALDI-MS), for direct in situ detection of Δ^9^-tetrahydrocannabinolic acid (∆^9^-THCA), cannabidiolic acid (CBDA), and other plant components. WALDI-MS (or SpiderMass) is designed for in vivo and real-time analysis under minimally invasive conditions of biological organisms [18,19]. The instrument achieves real-time metabolomic analysis through the combination of an IR-laser microprobe and a transfer tube on an MS instrument. The laser is used to achieve resonant excitation of water endogenous to the biological tissues and it promotes desorption/ionization with water acting as a matrix. In this study, we applied SpiderMass technology for the profiling and discriminating of∆^9^-THCA and CBDA directly in vivo in plants by real-time MS and MS/MS. We further investigated the different levels of cannabinoids across the plant and the possibility of classifying different cannabis cultivars based on their metabo-lipidomic profiles.

## 2. Result

### 2.1. Analysis of Cannabinoid Standards

**Figure 1:**
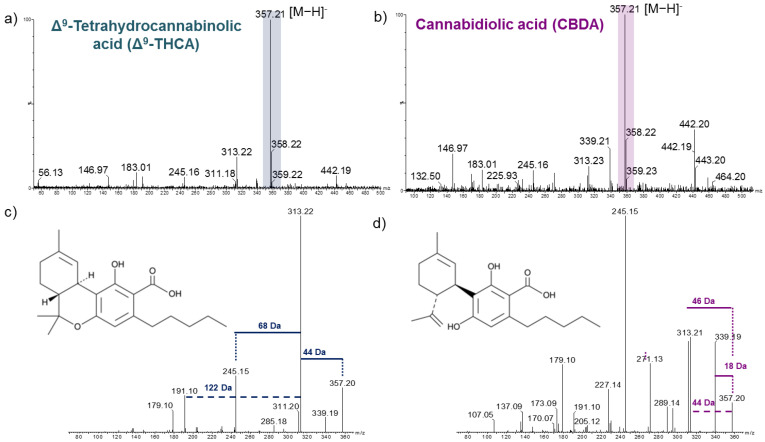
Cannabinoid mass and fragmentation negative ion mode spectra. (**a**) Δ^9^-tetrahydrocannabinolic acid (Δ^9^-THCA) and (**b**) cannabidiolic acid (CBDA). There is a clear molecular peak at *m*/*z* 357.2 corresponding to the [M−H]^−^. The fragmentation spectra of *m*/*z* 357.2 precursor ion for each cannabinoid are shown in (**c**,**d**).

To verify the detection of cannabinoids with SpiderMass, we first performed a direct analysis of Δ^9^-tetrahydrocannabinolic acid (Δ^9^-THCA) and cannabidiolic acid (CBDA) standards. The acids were analyzed in methanol with the addition of 1µL glycerol to stabilize the droplet by lowering the volatility and increasing the ionization efficiency. The MS spectra of the two standards in the negative ion mode are presented in Figure 1. The MS spectra contained the deprotonated Δ^9^-THCA (Figure 1a) and CBDA (Figure 1b) peak at *m*/*z* 357.2 as the base peak. As the two bioactive compounds are constitutional isomers, their molecular ion appears at the same *m*/*z*. Therefore, the only way to distinguish between the two is through MS/MS analysis. The MS^2^ spectra of the two standards are shown in Figure 1c,d. The fragmentation pattern of CBDA reveals a base peak at *m*/*z* 245.2. For Δ^9^-THCA, the most intense peak corresponds to the *m*/*z* 313.2 fragment, which is attributed to the neutral loss of 44 Da (CO_2_), resulting in the deprotonated Δ9-THC molecule [C_21_H_30_O_2_-H]^−^. However, Δ^9^-THCA also displays a peak at *m*/*z* 245.2, about 4–5 times less intense compared to CBDA. Both fragmentation spectra also indicate the presence of *m*/*z* 339.2 fragment corresponding to [M-H-H_2_O]^−^ and a subsequent loss of 28 Da (CO), generating the [C_21_H_28_O_2_-H]^−^ ion at *m*/*z* 311.2. There is also a subsequent transition from *m*/*z* 313.2 to *m*/*z* 245.2 by a neutral loss of 68 Da (C_5_H_8_) due to double hemolytic cleavage from the six membered rings. It has been previously demonstated in the literature that ∆^9^-THCA leads to an MS/MS spectrum dominated by a *m*/*z* 313.2 ion and *m*/*z* 245.2 for CBDA [17].

### 2.2. Detection of Acid Cannabinoids across the Plants

**Figure 2:**
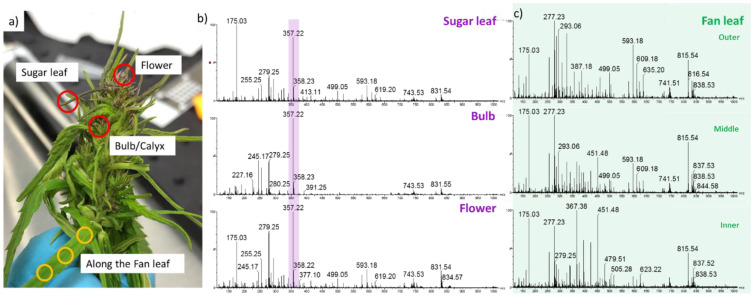
In vivo SpiderMass analysis on cannabis plants. (**a**) Photo of a cannabis plant with indicated plant organs and measurement points (red and yellow circles). (**b**) Examples of negative ion mode mass spectra from the sugar leaf, bulb, and flower. The highlighted area shows the *m*/*z* 357.2 ion that corresponds to the mixed signal of THCA/CBDA. (**c**) Examples of negative ion mode spectra recorded along the fan leaf (outer, middle, and inner).

**Figure 3:**
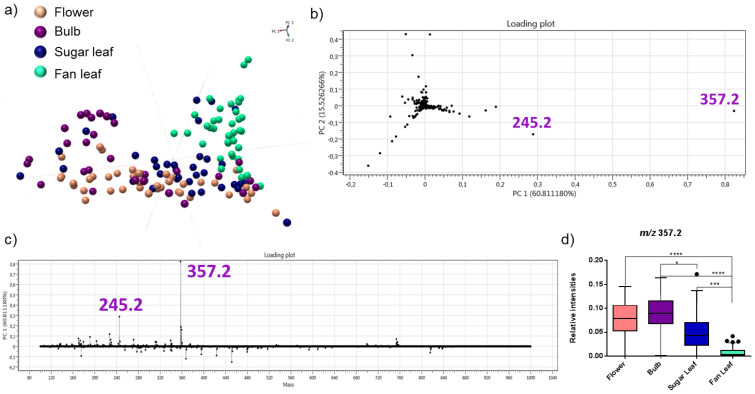
Multivariate statistical analysis of the molecular profiles from different plant organs combining all cultivars. (**a**) PCA plot and (**b**) PCA loading plot of 4 plant organs (flowers, bulbs, sugar leaves, and fan leaves) from all cultivars. (**c**) PC1 loading plot showing the highest variance between the flower (rose), bulb (purple), sugar leaf (blue), and fan leaf (green) due to the cannabinoid acid peak at *m*/*z* 357.2 and *m*/*z* 245.2 binned to 0.1 Da. (**d**) Relative intensity boxplots of the selected peaks showing the highest contribution to the variance of PC1. The intensities were normalized to the total ion chromatogram and represented as boxplots for each year with the Tukey method whisker definition. There was a significant difference for *m*/*z* 357.2 between the flower (**** *p* ≤ 0.0001), bulb (**** *p* ≤ 0.0001), and sugar leaves (*** *p* = 0.0005) compared to fan leaves. The significant difference is also observed between the bulb and the sugar leaves (* *p* = 0.0341).

SpiderMass analyses were performed on four different plant organs (Figure 2): flowers, bulb (the outermost whorl that forms the flower), sugar leaves (leaves developing around the flower), and three areas from the fan leaf (Figure 2a). In this way, we captured the molecular profiles across plant organs known to have the highest cannabinoid concentrations. Molecular differences were clearly visualized for each organ. An example is shown in Figure 2b,c. The flowers, bulb, and sugar leaves show relatively similar profiles in contrast to the fan leaves. In the MS spectra of the sugar leaves, bulb, and flowers, there is a very noticeable peak with high intensity detected at *m*/*z* 357.2. This peak corresponds to the deprotonated ∆^9^-THCA and CBDA isomers. On the other hand, only a low intense *m*/*z* 357.2 is observed across the fan leaves. The fan leaves, however, do display rich metabolic spectra within the *m*/*z* 200–900 range compared to the other parts of the plant. Additional principal component analysis (PCA) confirmed the spectral observations. The fan leaves (n = 39) showed a clear separation from the flowers (n = 42), bulbs (n = 28), and sugar leaves (n = 37) (Figure 3a). PCA 1 displayed 60.8% of variance mainly governed by the *m*/*z* 357.2 and the *m*/*z* 245.2 ions in the loading plot (Figure 3b) and loading-mass plot (Figure 3c). Further statistical tests (Figure 3d) showed a significant difference in the relative intensities comparing flowers (*p* ≤ 0.0001), bulbs (*p* ≤ 0.001), and sugar leaves (*p* = 0.0005) to fan leaves. A significant difference was also observed between bulb and sugar leaves (*p* = 0.0341).

### 2.3. Discrimination of Cannabis Cultivars

**Figure 4:**
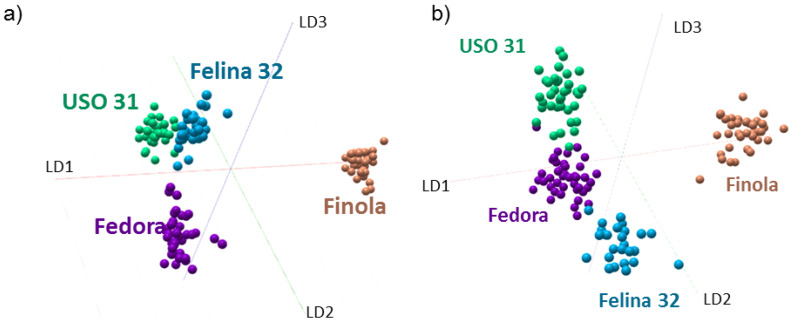
Multivariate statistical analysis of molecular profiles from different cannabis plant cultivars. LDA representation of the 4−class PCA−LDA including Finola, USO31, Felina 32, and Fedora in (**a**) positive and (**b**) negative ion modes. In both polarities the Finola subtype is well discriminated in LD1.

Four cannabis subtypes were used to create metabolic/lipid-based classifiers. The raw spectra of all four subtypes (Felina (n = 4), Fedora (n = 4), Finola (n = 4), and USO (n = 4)) and all regions (flower, bulb, sugar leaf, and fan leaf) were imported into a single classification model. The 3D LDA representation of the 4−-class PCA-LDA analysis of both positive and negative ion modes is shown in Figure 4a,b, respectively. Substantial discrimination was observed in LD1 for Finola compared to the remaining subtypes. The second component (LD2) discriminates Fedora in the positive and Felina in the negative ion mode. The loading plot of LD1 is shown in Supplementary Figure S1.

**Figure 5:**
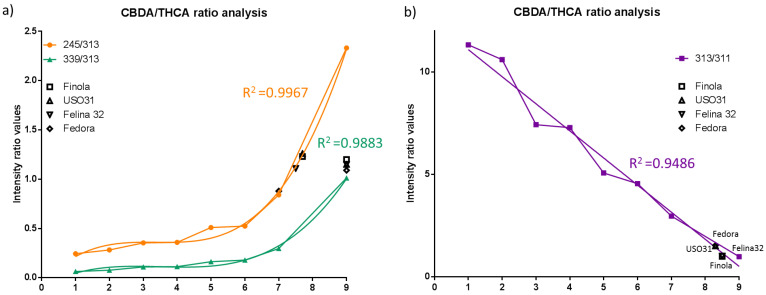
CBDA/∆^9^-THCA ratio analysis. Calibration curves calculated from different CBDA/∆^9^-THCA ratios. Ratios were calculated for several diagnostic MS/MS ions, mainly (**a**) *m*/*z* 245/313 and *m*/*z* 339/313 base peaks for CBDA > 1 ion and (**b**) 313/311 for ∆^9^-THCA > 1. The ratios were calculated from the MS/MS fragmentation patterns of flowers of each cultivar.

The discrimination between the different cultivars is mainly due to fatty acid, lipid, flavonoid, and plant wax ions (Supplementary Tables S1 and S2) and the weight of the *m*/*z* 357.2 ion is negligible. We further investigated the MS/MS fragmentation spectra from each flower to evaluate whether they do contain different levels of THCA and CBDA isomers (Supplementary Figure S2). This was achieved using the rapid and straightforward workflow based on the evaluation of the following diagnostic ions: *m*/*z* 245.2, 339.2, 313.2, and 311.2 [17]. Calibration curves were created for different ratios of CBDA/∆^9^-THCA standards (see Materials and Methods). The first point contained pure ∆^9^-THCA while the last point (9) contained pure CBDA (Figure 5). The calculated ratios for *m*/*z* 245/313 and 339/313 display a third order polynomial curve (Figure 5a), while the 313/311 ratio displays a linear regression fit (Figure 5b). The calculated R^2^ of each curve was >0.94. The same ratios were calculated for each cultivar and plotted on the graph. The calculated values are shown in Supplementary Table S3.

Additional analyses on the dried plants were performed using a validated HPLC-UV method for Fedora and Felina cultivars. The dosage results for THC, THCA, CBD, and CBDA are shown in Supplementary Table S4. The total CBDA in the flower was 1.193% and 1.622% for Fedora and Felina, respectively, while the total THCA was 0.049% and 0.066%, respectively.

## 3. Discussion

We demonstrated the possibility of analyzing cannabis plants directly in vivo using SpiderMass. As the analysis is mini-invasive it can be performed directly on plant organs. The analysis of each plant organ (flowers, bulb, sugar leaves, and fan leaves) resulted in a metabolic-rich spectrum. In the first instance, all the spectra from different cultivars were used to assess the presence of the cannabinoids. The multivariate statistical analysis immediately revealed the presence of *m*/*z* 357.2 attributed to THCA and CBDA in all cultivars but with a much higher intensity in the flowers, bulbs, and sugar leaves. There was a significant difference in relative intensities when comparing the flowers (*p* ≤ 0.0001), bulb (*p* ≤ 0.0001), sugar leaves (*p* = 0.0005) to fan leaves. This demonstrates the change in abundancies of cannabinoids along the cannabis plant and confirms that the highest phytocannabinoid concentrations are found in the flowers [20].

The different cultivars were discriminated among each other, through their molecular profiles and by the comparison of ∆^9^-THCA and CBDA ratios with MS/MS diagnostic ions. As both isomers share the same deprotonated molecular ion at *m*/*z* 357.2, they can only be discriminated by their fragmentation pattern. As proposed by previous works [17,21], Δ9-THCA showed a base peak at *m*/*z* 313.2, while it was *m*/*z* 245.2 for CBDA. In addition, Piccolella et al. [17] have described several ratios of other diagnostic ions, such as *m*/*z* ratio 339/313 (CBDA > 1) and *m*/*z* ratio 313/311 (∆^9^-THCA > 1) to be indicative of the presence of THCA and CBDA in samples. The most valuable information to discriminate the isomers is achieved when the parent ion fragmentation reaches about 70–75% (at 35−45 V CE using nitrogen gas). In our case, the parent ion fragmentation was reached by using CE = 25 V with argon gas. Indeed, the observation of diagnostic fragment ions with specific intensities is linked to their center-of-mass energy, which depends on the parent ion mass, the mass of the target gas for CID, and the used collision energy (inter quadrupole voltage difference) [22]. The same collision energy was then used throughout our analysis of standards and samples.

We further used the ratios of diagnostic ions to create three different standard calibration curves. The ratio of the *m*/*z* 339/313 increases, while the ratio of 313/311 decreases with a higher abundance of CBDA [17]. The ratios of each cultivar were then plotted onto the graphs. We clearly observed differences in the Fedora cultivar. The Fedora cultivar displays a lower abundance of CBDA compared to the Felina cultivar. The same results were observed with the HPLC-UV analysis. The SpiderMass ratio results cannot be directly compared with absolute quantitative analysis, as the sampling occurred only from a few µm and in vivo from fresh plants. It is also important to take into account the cultivation conditions (soil, climate) of the cultivar. Fedora, for example, displays lower CBDA content with SpiderMass and conventional HPLC-UV than expected from previous studies [23,24] and a higher THCA ratio with SpiderMass together with the USO31 cultivar.

It is not only the soil and climate but also the presence of certain terpenes, flavones, and other organic molecules that influence the production and ratios of THCA and CBDA present in different varieties [2,4,25,26]. For this reason, we also compared the global metabolic profile of each cultivar. The PCA-LDA analysis revealed a high degree of separation between each cultivar. Particularly, the Finola cultivar demonstrated a substantial discrimination between the varieties in both positive and negative ion modes. Even though Finola displayed higher CBDA and THCA ratios, the discrimination was mainly based on small metabolites, fatty acids, and lipid species present throughout the plant.

The above results demonstrate a high degree of separation for each cultivar either by the whole metabolic profile or through diagnostic MS/MS fragments. We can, therefore, foresee the methodology being adapted for further in vivo analysis of plant cultivars in the hemp industry and for the discrimination of cultivars in forensic analysis. These first results clearly demonstrate the sensitivity and versatility of SpiderMass technology.

To conclude, this first demonstration of SpiderMass in vivo on plants has shown the possibility in differentiating between THC- and CBD-acid cannabinoids in cannabis cultivars. Interestingly, our current method is rapid and does not require any sample preparation while retaining the spatial information. To further improve the separation of the THCA and CBDA isomers, without chromatographic separation, the use of ion mobility separation would be interesting to explore in the future [27,28]. The additional degree of separation would definitely improve the THCA and CBDA fragment ion calculations. Regardless, SpiderMass is, therefore, a sensitive and selective screening technique for both targeted and untargeted analysis of cannabis cultivars that can be used in the future for toxicology and forensic analysis.

## 4. Materials and Methods

### 4.1. Cannabinoid Standards

Δ9-tetrahydrocannabinolic acid (Δ9-THCA) and cannabidiolic acid (CBDA) standards at 1 mg/mL were purchased from LGC (Molsheim, France). The samples were stored at −20 °C. For HPLC/UV analysis, the water and methanol were purchased from Biosolve (Valkenswaard, The Netherlands), Dionex Ultimate 3000 RS from ThermoFisher (Merelbeke, Belgium), Restek Raptor ARC-18 column from Restek Bad (Homburg, Germany), Acetonitrile/0.1% formic acid from Fischer Chemical (Merelbeke, Belgium), ammonium formate from ChemLab, Zedelgem, Belgium. The THCA and CBDA standards used for the HPLC-UV method were purchased from Lipomed (Arlesheim, Switzerland), with THC from Cerilliant, Diegem, Belgium and CBD from LGC (Molsheim, France).

### 4.2. Cultivation of Cannabis

Four cannabis plants of each subtype or “cultivar” (USO, Finola, Fedora, and Felina) were provided by a local hemp producer from Luxemburg. All plants were cultivated outdoors and harvested on 11 July 2021. The samples were cooled at 5 °C to be preserved until analysis, which was achieved within the 48 h after the harvest.

### 4.3. SpiderMass and Data Analysis

The basic design of the instrument setup is described in detail elsewhere [18,19]. Briefly, the prototype was equipped with a fibered tunable wavelength laser system (Radiant version 1.0.1, OPOTEK Inc., Carlsbad, CA, USA) and pumped with 1.064 µm radiation delivered by a Q-switched 10 ns pulse width Nd:YAG laser (Quantel Laser, France). The infrared (IR)-laser microprobe was tuned (2.94 µm) to excite the most intense vibrational band of water (O-H). A 1 m biocompatible laser fiber with a 450 µm inner diameter (HP fiber, Infrared Fiber Systems, Silver Spring, MD, USA) was connected to the exit of the optical parametric oscillator (OPO) and focused to result in a 400–500 µm beam diameter. Tygon^®^ tubing (Akron, OH, USA) was used to aspirate the ablated material and was directly connected to the Q-TOF (Synapt G2s or Xevo, Waters, UK) mass spectrometer interface [29]. The resolution of the mass spectrometer was 18,000. With SpiderMass direct analysis no lock mass correction can be performed, resulting in a slight decrease in mass accuracy. All data were binned to 0.1 Da.

For the standards, the analysis was conducted from 1 µL of standard deposited with 1 µL of glycerol onto a Prosolia Omni TM glass slide. To create the calibration curves based on diagnostic MS/MS fragment ions, different CBDA/∆^9^-THCA ratios were used by increasing the number (100% ∆^9^-THCA, 2:8 CBDA: ∆^9^-THCA, 3:7 CBDA: ∆^9^-THCA, 4:6 CBDA: ∆^9^-THCA, 5:5 CBDA: ∆^9^-THCA, 6:4 CBDA: ∆^9^-THCA, 7:3 CBDA: ∆^9^-THCA, and 100% CBDA). In all cases, 1 µL of standard was deposited with 1 µL of glycerol.

For the plants, different regions were analyzed, including the flower, bulb, sugar leaves, and along the fan leaf, by directly placing the laser at the right focal distance from the laser probe. For each sample, the acquisition was composed of 10 laser shots consecutively fired (i.e., 1 s) repeated 3 times with a 10 s pause between each series. Ten laser shots resulted in one individual MS spectrum. The laser energy was fixed at 4 mJ/pulse and the spot size was 500 µm. The data were acquired in both polarities in the sensitivity mode in the *m*/*z* 100–2000 mass range. Four independent biological repetitions were realized for each cannabis plant subtype. The raw data were then imported into “Abstract Model Builder”—AMX (version 1.0.1972.0, Waters, Hungary). The classification model was built using individual spectra (on average 3 per sample) and by subjecting them to principal component analysis (PCA) and linear discriminant analysis (LDA). The classification model was built using a mass range of 100–1000 *m*/*z* with 0.1 binning, 1 × 10^3^ threshold intensity, applied normalization to the TIC, and background subtraction. A non-parametrical one-way ANOVA (Kruskal–Wallis) followed by Dunn’s test was performed to calculate the significant differences between the normalized intensities for discriminative ions using GraphPad Prism 5. The putative assignments of flavonoids, waxes, fatty acids, and lipids were made through the METLIN and ALEX123 databases [30,31].

The cannabinoid peaks were selected for MS/MS analysis with a 0.1 Da isolation window. MS/MS was performed with collision-induced dissociation (CID) using argon as the collision gas and a collision energy set to 25 V. The fragmentation spectra were then further used to evaluate the presence of CBDA and ∆^9^-THCA based on the ratios of neutral losses 18 Da/44 Da and 44 Da/46 Da. Relative abundances were calculated for the *m*/*z* 245.26 and 313.23 fragment ions for each cultivar based on the base peak from the fragmentation spectra.

### 4.4. HPLC-UV Analysis

Cannabinoid content was determined using a validated HPLC/UV method. In total, 500 to 1000 mg of the sample was added to 20 mL of ethanol, ultrasonicated for 15 min, and diluted in a 25/75 mixture of H_2_O/MeOH to obtain cannabinoid concentrations of approximately 1000 mg/L. Analyses were carried out on a reverse phase high-performance liquid chromatography system coupled to a UV detector (Dionex Ultimate 3000 RS HPLC/UV system). In total, 20 μL of the extracts were injected into the system. Chromatographic separation was achieved on a Restek Raptor ARC-18 column (100 mm × 3.0 mm × 1.8 μm). A photodiode array detector recording from 190 to 400 nm was used for detection and quantification. THCA and CBDA were quantified at 223 nm;THC and CBD were quantified at 209 nm. For chromatographic separation, we used a mixture of H_2_O, 5 mM ammonium formate/0.1% formic acid (eluent A), and acetonitrile/0.1% formic acid (eluant B). Isocratic conditions were applied for the separation (25% A and 75% B). The flow rate was at 1 mL/min and the total run time was 4.5 min. The oven temperature was set at 30 °C. A 6-point external calibration curve was established before analysis. The absolute amounts injected were 2, 4, 50, 100, 200, and 400 ng of each cannabinoid diluted in a 25/75 mixture of H_2_O/MeOH. Validation parameters included the determination of the linearity, intraday precision and accuracy, limit of detection (LOD), and lower limit of quantification (LLOQ). The LOD was defined as a signal to noise ratio equal or above three, and the LLOQ was defined as a signal to noise ratio equal or above 10. The acceptance criteria of the calibration curve were: coefficient of determination (r2) >0.99, resolution >1.5, accuracy bias <15% when compared to a control sample with known THC concentration, and relative standard deviation of the peak purity index for THCA and CBDA <5%.

## Data Availability

The data that support our findings in the study are available by contacting the correspondence author, I.F. The data are not publicly available due to privacy.

## Supplementary Materials

The following are available online at https://www.mdpi.com/article/10.3390/metabo12060480/s1, Figure S1: LD1 loading-mass spectra showing the discrimination between Finola and the rest of the cultivars, Figure S2: MS/MS fragmentation spectra from the flowers of each cannabis subtype, Table S1: Putative annotations of discriminative peaks between different cultivars in negative ion mode, Table S2: Putative annotations of discriminative peaks between different cultivars in positive ion mode, Table S3: Calculated relative abundances, standard deviation (σ), and coefficient of variance (CV) for *m*/*z* 339.2, 313.2, 311.2, and 245.2 ions for each cultivar, Table S4: Dosage results for THC, THCA, CBD, and CBDA in Fedora and Felina cultivars following LC-UV analysis.
