# Supplementary material for: Direct In Vivo Analysis of CBD- and THC-Acid Cannabinoids and Classification of Cannabis Cultivars Using SpiderMass
Source: Metabolites. 2022 May 26;12(6):480. doi: 10.3390/metabo12060480 (PMC9227750; doi:10.3390/metabo12060480)
Supplement: Supplementary file 1 [file metabolites-12-00480-s001.zip › metabolites-1551550-supplementary.pdf]

Supplementary Materials

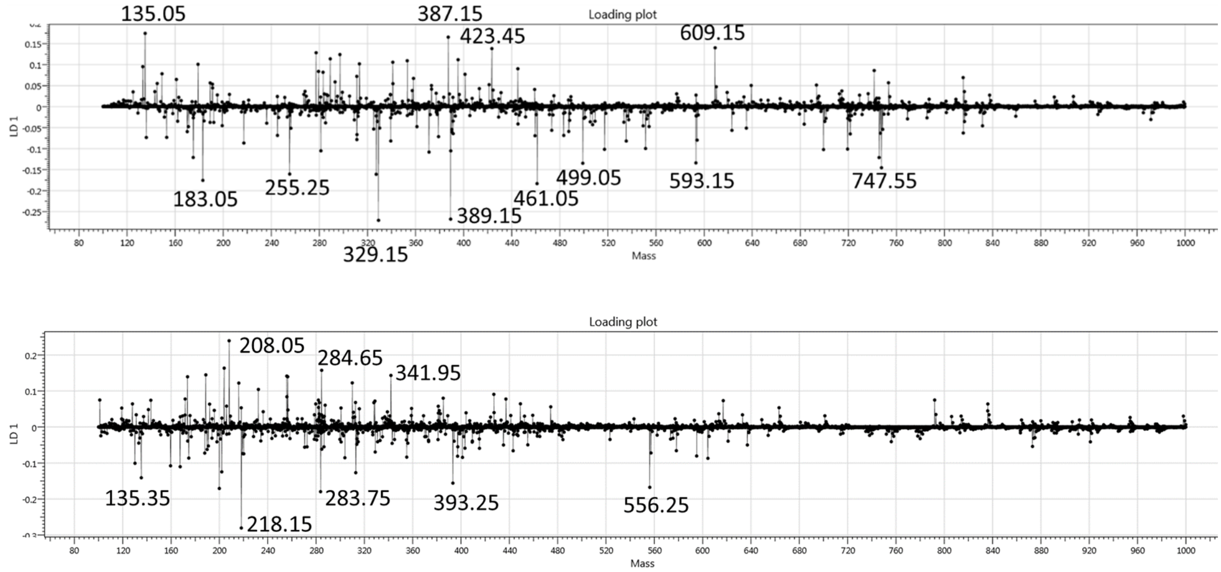

Supplemental Figure S1. LD1 loading mass spectra showing the discrimination between the Finola LD1 + and the rest of the Cannabis subtypes LD1- in negative (top) and positive (bottom) ion mode. The most discriminative peaks are indicated in the loading spectra.

-

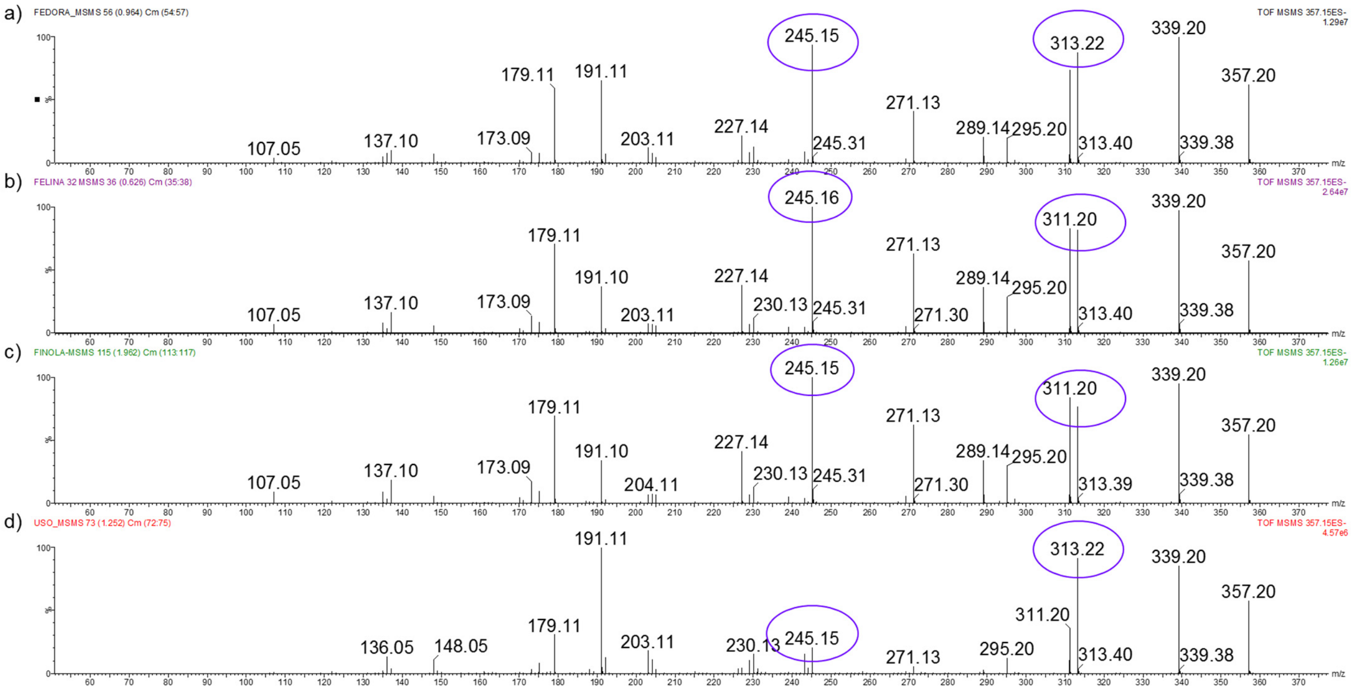

Supplemental Figure S2 MS/MS fragmentation spectra from the flowers of each cannabis subtype (**a**) Fedora, (**b**) Felina, (**c**) Finola and (**d**) USO. The highlighted fragments correspond to the indicated ions for the workflow for rapid and straightforward discrimination of acid phytocannabinoid isomers. The fragments indicated the presence of THC- and CBD- acid type cannabinoids.

Table S1. Putative annotations of the discriminative molecular species from different cultivars in negative ion mode. The annotations were made using Metlin and Alex123 databases with 0.1 Da tolerance.

m/z ion in negative ion mode	Putative annotation
**m/z 183.05**	Non-esterified FA (10:2;1) $[M-H]^-$
**m/z 255.25**	Non-esterified FA (16:0) $[M-H]^-$
**m/z 423.45**	♦(R)-nonacosan-10-ol $[M-H]^-$<br>♦Nonacosan-13-ol$[M-H]^-$
**m/z 387.15**	3,5-Dihydroxy-6,7,8-trimethoxy-3',4'-methylenedioxyflavone [M-H]-
**m/z 499.05**	Saflufenacil $[M-H]^-$ (herbacide)
**m/z 593.15**	*Cyanidin 3-neohesperidoside $[M-H]^-$<br>*Pelargonidin 3,7-di-glucoside $[M-H]^-$<br>*Saponarin $[M-H]^-$<br>*Kaempferol-3-O-glucosyl(1-2) rhamnoside $[M-H]^-$
**m/z 609.15**	*Cyanidin 3-galactoside-5-glucoside $[M-H]^-$<br>*Glucosylvitexin $[M-H]^-$<br>*Flavocannabiside $[M-H]^-$<br>*Cyanidin 3-sophoroside $[M-H]^-$
**m/z 747.55**	PG(O-35:1) $[M-H]^-$, PG(P-35:0) $[M-H]^-$, PG(34:1) $[M-H]^-$

* A couple of examples of the potential flavonoids

♦ A couple of examples of potential plant waxes

Table S2. Putative annotations of the discriminative molecular species from different cultivars in positive ion mode. The annotations were made using Metlin and Alex123 databases with 0.1 Da tolerance.

m/z ion in positive ion mode	Putative annotation
**m/z 218.15**	Acylcarnitine FA (3:0)$[M+H]^+$
	Non-esterified FA (11:1;1) $[M+ NH_4]^+$
	Non-esterified FA (12:0) $[M+ NH_4]^+$
**m/z 393.25**	LPA O-16:2 $[M+H]^+$
	LSM 13:2;2 $[M+H]^+$
**m/z 556.25**	TAG 30:16;1$[M+NH_4]^+$
	LPI 14:3 $[M+NH_4]^+$
	LPS 21:6 $[M+H]^+$
	LPS 19:3 $[M+Na]^+$
	TAG 31:15 $[M+NH_4]^+$
	LPE 24:5 $[M+H]^+$

Table S3. Calculated relative abundances, standard deviation (σ), and coefficient of variance (CV) for m/z 339.2, 313.2, 311.2, and 245.2 ions for each cultivar. Calculated ratios for 18Da/44Da and 44Da/46Da neutral losses for each cultivar.

Relative abundances (N=4)		m/z 339.2	σ	CV	m/z 313.2	σ	CV	m/z 311.2	σ	CV	m/z 245.2	σ	CV
Cultivar	Finola	0.968	0.055	0.06	0.822	0.017	0.02	0.821	0.044	0.05	0.940	0.066	0.07
	USO	0.889	0.048	0.05	0.748	0.099	0.13	0.478	0.036	0.07	1.000	0.000	0.00
	Felina	0.996	0.008	0.01	0.825	0.104	0.13	0.792	0.126	0.16	0.963	0.030	0.03
	Fedora	0.975	0.031	0.03	0.935	0.075	0.08	0.632	0.038	0.06	0.792	0.016	0.03

		245/313	18Da/44Da	44Da/46Da
Cultivar	Finola	1.23	1.20	1.00
	USO	1.26	1.15	1.56
	Felina	1.11	1.15	1.06
	Fedora	0.87	1.09	1.54

Table S4. Dosage results for THC, THCA, CBD and CBDA in Fedora and Felina cultivars following LC-UV analysis. THCA and CBDA slowly decay to THC and CBD. To obtain the total amount of THC+THCA and CBD+CBDA were added respectively, including the conversion factor which is 0.877 for both compounds.

FEDORA	TOX22_0474 flower (%)	TOX22_0474 leaf (%)	Ratio flower/leaf
THC	0.012	0.003	4.00
THCA	0.035	0.022	1.59
**THCA total**	**0.049**	**0.025**	1.92
CBD	0.074	0.029	2.55
CBDA	1.109	0.652	1.70
**CBDA total**	**1.193**	**0.685**	1.74

FELINA	TOX22_0475 flower (%)	TOX22_0475 leaf (%)	Ratio flower/leaf
THC	0.010	0.003	3.33
THCA	0.055	0.032	1.72
**THCA total**	**0.066**	**0.035**	1.87
CBD	0.053	0.022	2.41
CBDA	1.562	0.934	1.67
**CBDA total**	**1.622**	**0.959**	1.69
